# Ovine serum biomarkers of early and late phase scrapie

**DOI:** 10.1186/1746-6148-6-49

**Published:** 2010-11-02

**Authors:** Isabelle Batxelli-Molina, Nicolas Salvetat, Olivier Andréoletti, Luc Guerrier, Guillaume Vicat, Franck Molina, Chantal Mourton-Gilles

**Affiliations:** 1 Complex system modeling and engineering for diagnosis, SysDiag - UMR 3145 CNRS/Bio-Rad, 34184 Montpellier Cedex 4, France; 2 INRA-ENVT Toulouse, UMR1225, 31076 Toulouse, France; 3 Bio-Rad Laboratories, 92430 Marnes-la-Coquette, France

## Abstract

**Background:**

Transmissible spongiform encephalopathies are fatal neurodegenerative disease occurring in animals and humans for which no *ante-mortem *diagnostic test in biological fluids is available. In such pathologies, detection of the pathological form of the prion protein (i.e., the causative factor) in blood is difficult and therefore identification of new biomarkers implicated in the pathway of prion infection is relevant.

**Methods:**

In this study we used the SELDI-TOF MS technology to analyze a large number of serum samples from control sheep and animals with early phase or late phase scrapie. A few potential low molecular weight biomarkers were selected by statistical methods and, after a training analysis, a protein signature pattern, which discriminates between early phase scrapie samples and control sera was identified.

**Results:**

The combination of early phase biomarkers showed a sensitivity of 87% and specificity of 90% for all studied sheep in the early stage of the disease. One of these potential biomarkers was identified and validated in a SELDI-TOF MS kinetic study of sera from Syrian hamsters infected by scrapie, by western blot analysis and ELISA quantitation.

**Conclusions:**

Differential protein expression profiling allows establishing a TSE diagnostic in scrapie sheep, in the early phase of the disease. Some proteic differences observed in scrapie sheep exist in infected hamsters. Further studies are being performed to identify all the discriminant biomarkers of interest and to test our potential markers in a new cohort of animals.

## Background

Scrapie is a well-known prion-associated sheep encephalopathy that was identified in the XVIII century. Scrapie is a fatal neurodegenerative disease and related forms affect also humans (i.e., Creutzfeldt-Jakob disease) and cattle (i.e., bovine spongiform encephalopathy). It is characterized by accumulation in the central nervous system of a pathological agent, the prion protein (PrP^Sc^) [1], which differs from the endogenous normal form (PrP^c^) in conformational changes, partial resistance to proteolytic degradation and insolubility in the presence of detergents [2,3]. Scrapie is a good model to study transmissible spongiform encephalopathies (TSEs) since the disease is related to genetic factors. The natural occurrence of scrapie is associated with the *PRNP *genotype at positions 136, 154, 171 [4-6]. Specifically, infected animals present the homozygous PrP^VRQ^/PrP^VRQ ^genotype, whereas healthy ones the homozygous PrP^ARR^/PrP^ARR ^genotype. The incidence of the pathology is predicted as 100% for PrP^VRQ^/PrP^VRQ ^animals whilst PrP^ARR^/PrP^ARR ^animals are considered resistant [7-10]. However, few cases of scrapie in PrP^ARR^/PrP^ARR ^sheep have been reported with biochemical and transmission characteristics similar to those of classic scrapie, although PrP^Sc ^was associated with lower proteinase K-resistance [11,12]. In contrast to BSE, scrapie is associated with wide PrP^Sc ^dissemination in many non-neural tissues including the lymphoreticular system, the kidney and the placenta [13]. The incubation period of the disease is long and silent (i.e., early, replicative phase of PrP^Sc^) and clinical symptoms appear in sheep aged from twelve to fifteen months (i.e., late, neuroinvasion phase of the disease). PrP^Sc ^can be detected in PrP^VRQ^/PrP^VRQ ^sheep two months after infection [14]. Between three to six months after infection, the pathological agent is detected essentially in lymphoid formations associated to the gastrointestinal tract. From six to nine months the secondary lymphoïd organs are also infected and finally at the tenth month after infection the central nervous system is affected [6,10,15].

At the moment, unambiguous diagnosis is only possible *post-mortem *and it is based on the detection of PrP^Sc ^after proteolytic digestion. A complementary diagnostic evaluation can be performed by immunohistochemistry, western blot or ELISA but none of these methods can detect scrapie during the incubation period without autopsy. Since PrP^Sc ^can accumulate in lymphoid tissues before spreading to the nervous system and this accumulation can be very extensive, some authors have proposed using this tissue for the *in vivo *and *post-mortem *diagnosis of scrapie [16-20]. However, the sensitivity of this methodology is not well characterised because the magnitude and duration of lymphoid tissue involvement can vary considerably [21].

The diagnosis of TSEs during the early phase by a rapid test performed in blood is thus required because of the existence of the variant Creutzfeldt-Jakob disease in humans and its possible iatrogenic transmission by blood [22]. Currently, several candidate *ante-mortem *biomarkers from serum, cerebrospinal fluid or tissue have been identified (i.e.,14.3.3 protein [23], NSE [24,25], S100B proteins [26,27], tau proteins [28-31], apolipoprotein E [32], C reactive protein and IL-6 [33], cystatin C [34], EDRF [35,36]), but none of them is specific or sensitive enough to be used in a routine diagnostic test. The only useful marker for diagnostic tools still is PrP^Sc ^[37,38], but its application in an *ante-mortem *test of prion disease in animals and humans, as proposed by Castilla and collaborators [39,40], with the "Protein Misfolding Cyclic Amplification", is still difficult and no significant results are available yet. A new detection method of disease-associated multimeric forms of the prion protein in plasma of prion-affected hamsters and sheep is called 'Multimer Detection System' [41]. The first results provided have now to be confirmed. Recently, the PrP^Sc ^has been detected in blood from sheep infected with scrapie and bovine spongiform encephalopathy [42] and in milk from ewes incubating natural scrapie [43].

We therefore decided to identify blood proteins that could be involved in scrapie development by analyzing a collection of serum samples from healthy and diseased sheep using proteomic tools. The classical proteomic tool, the bi-dimensional gel electrophoresis (2-DE) has been developed 25 years ago. Although this technique permits a high resolution separation of proteins, sensitivity and reproducibility of these experiments are not optimal to analyze hundred of highly variable animal samples. Moreover, 2-DE allows the separation of thousands of proteins but rare proteins are often not detected and low molecular weight proteins are not resolved. On the other hand, Surface Enhanced Laser Desorption/Ionization-time of flight-mass spectrometry (SELDI-TOF MS, Ciphergen Biosystems, Fremont, CA, USA), by combining selective protein binding with sensitive and quantitative mass detection, allows the evaluation of protein profiles in a short time from a large number of samples to identify putative biological markers [44-46]. SELDI-TOF MS was thus used to characterize the protein profile of sera from sheep at early (replication) and late (neuroinvasion) stages of scrapie. This analysis allowed us to identify a combination of biomarkers that discriminate between early phase (EP) and late phase (LP) of infection. We then validated these findings by assessing the appearance of differential markers in hamsters infected with the 263 K scrapie strain, a model of prion infection with shorter kinetics compared to sheep.

## Methods

### Scrapie evaluation and serum sampling

A total of 163 serum samples of sheep from a naturally scrapie-affected Romanov flock [14] were used in this study. Blood samples were collected at the veterinary laboratory of Toulouse (INRA, France). Sheep were classified according to their genotype and histopathological characteristics as shown in Table 1. The sheep in the early phase of infection (EP) are 7 to 10 months/old, they were asymptomatic. The sheep in the late phase of infection (LP) are 13 to 19 months/old and developed clinical symptoms. We included 78% of females and 22% of males (representative of gender proportion in this sheep population). The sheep presenting the homozygous PrP^ARR^/PrP^ARR ^genotype are 7 to 94 months/old. Serum from 3 VRQ/VRQ Cheviot TSE free sheep (Arthur Rickwood, UK) was collected and included in the study as a negative control. This flock is the only source in Europe of sheep free of classical scrapie.

**Table 1:** Clinical characteristics of control and diseased sheep

Histopathology	Genotype	Age in months	Sex	Strain	Number
Controls	ARR/ARR	7 to 95	Male and Female	Romanov	65
Late phase scrapie (LP)	VRQ/VRQ	13 to 19	Male and Female	Romanov	43
Early phase scrapie (EP)	VRQ/VRQ	7 to 10	Male and Female	Romanov	55
					total 163

### Infection of Syrian hamsters with the 263 K scrapie strain

Infected animals were housed in controlled facilities fully compliant with the European policy on use of Laboratory Animals (agreement n°A3 4-175-28). The European guidelines (EC-86/609) for animal care and experimentations were followed throughout the duration of the study. They were identified by electronic chips. Ten age-matched Syrian hamsters were injected intra-peritoneally with 5 microliters (μL) (670 micrograms) of homogenate of brain infected by the 263 K scrapie strain diluted into 95 μL of phosphate buffer saline. 200 mg of infected brain has been crushed into 1.5 milliliters (mL) of 20% sucrose. Serum samples were taken at day 0, 29, 57, 106 and 150 post-infection. Each hamster was sacrificed at the end of the kinetics when it had lost ≈ 30% of its body weight and was no longer able to remain upright and feed by itself [47].

### Serum protein fractionation

Blood was collected in dry tubes; coagulation was allowed by incubating samples at 37°C for 30 minutes. Following centrifugation, sera were immediately stored at -80°C.

Samples were pre-fractionated by anion exchange chromatography according to their charge characteristics, using the "Expression Difference Mapping Kit-Serum Fractionation" from Ciphergen Biosystems. Briefly, 20 μL of serum were added to each well of a 96-well culture microplate and proteins were denatured by addition of 30 μL of 9 M urea, 2% CHAPS, 50 mM Tris-HCl pH9. Then, each denatured aliquot was transferred into a filtration microplate containing in each well Q Hyper D F sorbent beads that had been previously rehydrated with 200 μL of 50 mM Tris-HCl pH9 (three times) and equilibrated with 1 M urea, 0.2% CHAPS, 50 mM Tris-HCl, pH9 (three times). In order to bind sample to sorbent, 50 μL of 1 M urea, 0.2% CHAPS, 50 mM Tris-HCl, pH9 buffer were added to each well and mixed for 30 minutes at 4°C. To collect th e six fractions we added sequentially 200 μL of 50 mM Tris-HCl with 0.1% OGP, pH9 (Fraction 1, F1) onto each well of the filtration microplate, then 200 μL of 50 mM Hepes with 0.1% OGP, pH7 (F2), then 200 μL of 100 mM sodium acetate with 0.1% OGP pH5 (F3), then 200 μL of 100 mM sodium acetate with 0.1% OGP pH4 (F4), then 200 μL of 50 mM sodium citrate with 0.1% OGP, pH3 (F5) and finally 200 μL of 33.3% isopropanol, 16.7% acetonitrile, 0.1% trifluoroacetic acid (F6, organic wash). Fractions were stored at -80°C.

### SELDI-TOF MS analysis of sera

ProteinChip^® ^CM10 (weak cation exchanger, Ciphergen Biosystems) were pre-treated with 5 μL of 0.1 M sodium acetate, pH4 twice. Five μL of each fraction were diluted in 5 μL of 0.1 M sodium acetate pH4 for use with weak cation exchanger (CM10) 8-spot protein chip arrays and incubated on a shaker in a humidified chamber at room temperature for 30 minutes. Spots were washed with 5 μL of 0.1 M sodium acetate pH4 twice for 5 minutes, followed by a quick rinse in de-ionized H_2_O. After air-drying, a sinapinic acid solution (70% acetonitrile, 0.1% trifluoroacetic acid) (SPA, Ciphergen Biosystems), prepared according to the manufacturer's instructions, was added to each spot. Arrays were analyzed with a PBS-II mass reader (Ciphergen Biosystems) using the SELDI 3.2.1 software (Ciphergen Biosystems). We performed the data acquisition of low molecular weight proteins by detecting the optimized size range between 2 and 20 kDaltons (kDa) with a maximum size of 30 kDa. Data were collected by averaging 60 laser shots with an intensity of 260 arbitrary units. The mass-to-charge ratio (m/z) of each protein captured on the array surface was determined according to externally calibrated standards (Ciphergen Biosystems): Hirudin BHVK (7034 Da), bovine Cytochrome C (12 230 Da), equine Myoglobin (16 951 Da) and bovine Carbonic Anhydrase (29 023 Da).

### Biostatistics analysis

#### Signal analysis

All spectra were compiled and qualified mass peaks (signal-to-noise ratio > 5) with mass-to-charge ratios (m/z) between 2 kDa and 30 kDa were auto-detected. Peaks clusters were completed using second-pass peak selection (signal-to-noise > 2) within 0.3% mass windows and estimated peaks were added. To avoid matrix interference, we removed all signals below 2 kDa and peaks intensities were normalized to the total ion current of m/z between 2 kDa to 20 kDa. Analyses were performed using the Protein Chip Software 3.2.1 (Ciphergen Biosystems).

#### Differential analysis of peak intensities

All normalized spectra were exported into an expression matrix N × M where N represents the mass peak and M the serum sample; the relative intensity of each peak is available. Peaks were selected by their statistical significance using the "Significance Analysis of Microarray" method (SAM) [48], a parametric method based on a modified Student's *t*-test currently used in genomic analysis. Significant peaks were selected when their score deviated from the average score obtained after 2000 permutations of matrix. The accuracy of markers and their discriminatory power were evaluated through Receiving Operating Characteristics (ROC) curves analysis [49]. ROC curves are graphical visualizations of the reciprocal relation between sensitivity (Se) and specificity (Sp) of a test for various values. The Area Under the Curve (AUC) is a good evaluation of the combination of Se and Sp for a given test.

#### Statistical validation

The best markers were combined to increase their Se and Sp with the mROC program [50] and with two supervised learning algorithms, AdaBoost [51] and Support Vector Machine (SVM) [52,53]. The mROC program calculated the linear combination which maximized the area under the ROC curve for all selected variables (peaks). The equation for the respective combination was provided and could be used as a new virtual marker. For a marker combination and for a sample selected, the cut-off was the resulting value of the linear equation corresponding and calculated by the mROC program: Cut-off = a × Marker1 + b × Marker2 + c × Marker3, where a, b and c are coefficients. For this approach, data were previously transformed using the Box-Cox transformation to ensure a normal distribution [54].

The AdaBoost algorithm, short for Adaptive Boosting, is a machine learning algorithm formulated by Yoav Freund and Robert Schapire [51]. It is a meta-algorithm and can be used in conjunction with weak learning algorithms (as decision tree) to improve their performance. In addition, it is less susceptible to the overfitting problem than most learning algorithms. SVM has been recognized as the most powerful classifier in various applications of pattern classification. For binary classification, it performs classification tasks by constructing hyperplanes in a multidimensional space (via a kernel function) that separate two classes of data with the maximum margin.

To estimate the errors prediction of these classifiers, we used the 10-fold cross-validation method. To avoid over-fitting problems and to reduce variance, we repeated this 10-fold cross-validation procedure 10 times. For this approach, we used the "ipred package" and the "e1071-package" of the R software [55].

The *p *values were calculated by one way ANOVA or Wilcoxon test with Kaleidagraph 4.0 software.

### SELDI peak identification

The libraries of 48 sorbents were obtained from Sigma Aldrich (St Louis, MO, USA) and from Bio-Rad Laboratories (Hercules, CA, USA), as well as materials for electrophoresis such as plates and reagents.

#### Chromatography purification of the 12 030 Da (S10) biomarker from crude sample "F6"

After screening the libraries of 48 sorbents (10 μL each) on a NUNC SilentScreen 96-filter plate at two different pH binding conditions (5 and 8), two complementary sorbents were chosen to selectively interact at pH8 with either the 12 030 Da target (DEAE-Macroprep from Bio-Rad) or with the target impurities (immobilized arginine from Sigma Aldrich).

Screening was monitored by SELDI-TOF MS on CM10 arrays.

First, 500 μL of F6 sample diluted ten times in binding buffer (0.1 M Tris-HCl pH8, 0.15 M sodium chloride) were incubated in a Supelco spin column with 1 mL of arginine sorbent. Non-retained or flow-through fraction (FT), containing the 12 030 Da target, but now free of key impurities, was obtained by centrifugation (500 g for 5 minutes). Then, the FT fraction (5 mL) was poured in a 15 mL Falcon tube and 10 μL of DEAE-Macropep sorbent was added. Incubation was performed by gentle vertical stirring at room temperature for one hour. After incubation, FT fractions were centrifuged (1000 g for 10 minutes), beads transferred in a 500 μL microtube, extensively washed with 500 μL of binding buffer and the enriched 12 030 Da biomarker was sequentially eluted with 25 μl of the following eluents: a) 4.5 M urea, 1% CHAPS; b) 9 M urea, 2% CHAPS; c) 9 M urea, 2% CHAPS, 2.4% ammonium hydroxide. Elution was monitored by SELDI-TOF MS on CM10 arrays, and the most enriched 12 030 Da fraction (c) selected for final purification on SDS-PAGE after pH neutralization with acetic acid.

#### Preparative final purification of 12 030 Da marker by SDS-PAGE

25 μL of each enriched sample were mixed with 25 μL of Laemmli buffer (4% SDS, 20% glycerol, without reducing agent, 0.004% bromophenol blue and 0.125 M Tris-HCl, pH approx. 6.8) from Bio-Rad. The mixture was heated in boiling water for 2 minutes and immediately loaded on the gel. The SDS-PAGE gel was composed of a stacking gel (125 mM Tris-HCl, pH6.8, 0.1% SDS) with a large-pore polyacrylamide gel (4%) cast over the resolving gel (4-20% acrylamide gradient in 375 mM Tris-HCl, pH8.8, 0.1% SDS buffer). The cathodic and anodic compartments were filled with Tris-glycine buffer, pH8.3, containing 0.1% SDS. The electrophoretic run was done at 100 V until the dye front reached the bottom of the gel. Staining and de-staining were performed using the Colloidal Coomassie Blue staining kit from Invitrogen (Carlsbad, CA, USA). Putative blue bands were excised and split in two. The smallest part (one fourth) was to confirm the presence of the 12 030 Da biomarker by SELDI-TOF MS (NP20 array), after protein extraction with a solution of formic acid (5 vol)-acetonitrile (2.5 vol)-2-propanol (1.5 vol)-water (1 vol) for 2 hours at room temperature. The other part (three fourth) was trypsinized for protein identification by Liquid Chromatography MS/MS (LC-MS/MS) (see details in the corresponding section).

#### Analysis of crude and purified fractions by SELDI-TOF MS

After protein extraction, fractions at appropriate concentration, *i.e*. 0.02 μg/μL, were deposited upon ProteinChip^® ^array surfaces, using a Bioprocessor device. Two types of arrays were selected: CM10 (weak cation exchanger) and NP20 (silica surface used in Matrix Assisted Laser Desorption Ionization time of flight, MALDI-TOF mode). Each array contained eight distinct spots over which the adsorption of protein could be performed. After applying the samples, the chip surfaces were washed to remove non-associated protein (only for CM10 arrays) and then dried and prepared for analysis after application of 1 μL of energy adsorbing matrix solution composed of a saturated solution of sinapinic acid in 50% acetonitrile and 0.5% trifluoroacetic acid. All arrays were then analyzed with a PCS 4000 ProteinChip^® ^MS reader. The instrument was used in a positive ion mode, with an ion acceleration potential of 20 kVolts and a detector gain voltage of 2 kVolts. The mass range investigated was from 3 kDa to 20 kDa. The laser intensity was set between 200 and 250 units according to the sample tested. The instrument was mass calibrated with a kit of standard mass mixture "All-in-1 protein standard" (Bio-Rad).

#### Protein identification by LC-MS/MS

Putative blue bands manually excised from the gel were sent to the Functional Proteomic Platform in Montpellier (INRA, France) and protein identification was done according to a standard operating procedure. Tryptic peptides were analyzed by an ESI-Ion Trap mass spectrometer (Esquire HCT; Bruker Daltonik GmbH, Bremen, Germany), interfaced with an HPLC-Chip system (Agilent Technologies, Palo Alto, CA). A sample volume of 2 μl was loaded onto a C-18 enrichment cartridge (40 nL) with a flow rate of 0.3 μl/min of 0.1% (v/v) formic acid. After pre-concentration and clean-up, peptides were separated in the column (HPLC-Chip C18, 5 μm, 75 μmx43 mm, 40 nL enrichment column; Agilent Technologies, Palo Alto, CA) at a flow rate of 4 μl/min using a gradient of 3% to 80% (v/v) acetonitrile in 15 minutes (0.1% [v/v] formic acid). Peptides were eluted into the High Capacity ion Trap (Esquire HCT; Bruker Daltonik GmbH, Bremen, Germany). Capillary voltage was 1.5-2 kVolts in the positive ion mode and a dry gas flow rate of 4.5 L/minute with a temperature of 250 °C was used. The first full-scan mass spectrum was measured for a range from 310 to 1800 m/z. The second scan was done to exactly measure the M_r _of the three major ions with higher resolution and the third scan to measure the collision-induced MS/MS spectrum of the selected ions. Theorical peptide values were obtained from UniProt database [56], the corresponding access number of ovine transthyretin is P12303.

## Results

### Reproducibility study

**Figure 1:**
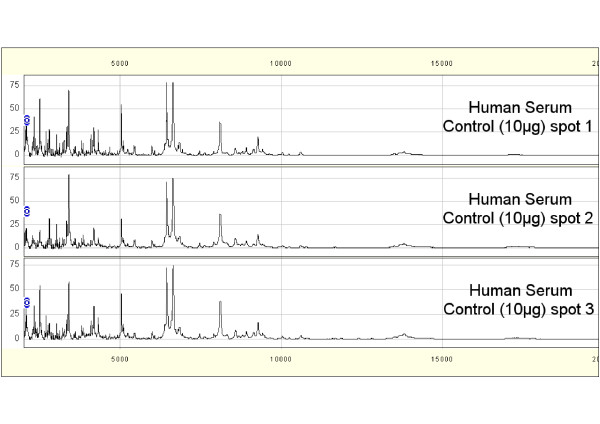
**Reproducibility of SELDI-TOF MS analysis using a human serum control**. A human serum control was fractionated three times on consecutive days and then the organic fraction F6 spotted and analyzed by SELDI-TOF MS in the range of 2-20 kDa on the same day.

First, to evaluate the reproducibility of SELDI-TOF MS analyses, 10 μg of proteins from a human serum control (Ciphergen Biosystems) were spotted onto three CM10 ProteinChip^® ^arrays at the same time. The mass spectra were analyzed in the 2-20 kDa range by SELDI-TOF MS under identical conditions in the same day (Figure 1). A range of 120 to 130 peaks were found in the three experiments. The peak number variations detected in the 2-20 kDa range (signal/noise ratio of at least 5) among samples were 7.69% (10/130) and the coefficient of variation of peak intensities was lower than 25%. The mass accuracy for such molecular weight was comprised between 0.1 and 0.2%. This test was performed before the analysis of sheep and hamster samples.

### Detection by SELDI-TOF MS of proteins which are differentially expressed in scrapie

**Figure 2:**
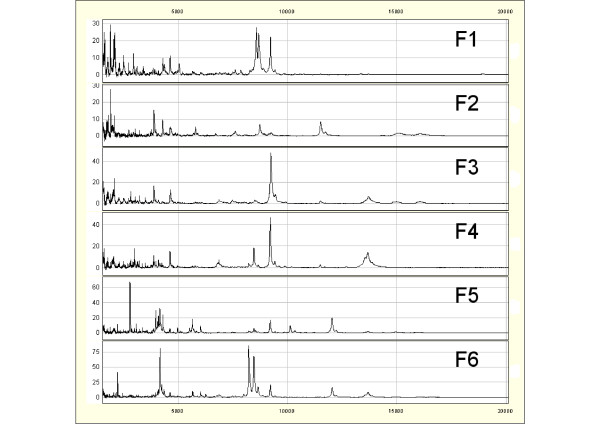
**Representative fractionated serum SELDI-TOF MS protein profiles of a PRP^ARR/ARR ^sheep**. F1 (pH9), F2 (pH7), F3 (pH5), F4 (pH4), F5 (pH3), F6 (organic wash).

To detect biomarkers characteristic of the EP and LP of scrapie in sheep, we carried out a SELDI-TOF MS analysis of the profiles of proteins included in the 2-20 kDa range in the anion-exchange fractions (at pH9, pH7, pH5, pH4 and pH3 and the organic wash) obtained from serum samples of sheep with (EP or LP) scrapie and healthy controls (Table 1) (see Figure 2 for representative PrP^ARR^/PrP^ARR ^sheep protein profiles of the different fractions). The majority of peaks were found to have a mass smaller than 10 kDa and few peaks were detected in the high mass molecular weight, above 20 kDa (data not shown). A total of 95 qualified mass peaks were detected by the Ciphergen Biosystems Software 3.2.1 for all spectra and were analyzed in an expression matrix. Differential analysis of peak intensities was conducted using the SAM method (Table 2).

**Table 2:** List of the 15 biomarkers that were differentially expressed in scrapie sheep in comparison to control animals

Markers	Mass (Da)	Fraction	Variation	Healthy (n = 65) *vs *EP (n = 55)				Healthy (n = 65) *vs *LP (n = 43)			
				FDR (%)	SAM	FC	AUC	FDR (%)	SAM	FC	AUC
S1	4030	F4	over	0,00	4,72	1,81	0,771	13,01	1,44	1,44	0,617
S2	4250	F1	under	0,00	-3,56	0,53	0,735	ND	ND	ND	ND
S3	7475	F3	over	0,00	3,39	1,75	0,741	ND	ND	ND	ND
S4	9395	F3	over	0,00	4,21	1,89	0,717	ND	ND	ND	ND
S5	3895	F6	under	ND	ND	ND	ND	15,34	-1,99	0,38	0,702
S6	7690	F6	over	ND	ND	ND	ND	0,00	2,31	1,26	0,659
S7	9425	F6	over	ND	ND	ND	ND	0,00	3,07	1,49	0,693
S8	27450	F6	over	ND	ND	ND	ND	4,77	2,20	1,39	0,672
S9	13670	F5	over	ND	ND	ND	ND	0,00	3,15	1,39	0,683
S10	12030	F6	under	ND	ND	ND	ND	10,74	-2,91	0,79	0,625
S11	8350	F6	over	ND	ND	ND	ND	4,77	2,12	1,19	0,648
S12	7555	F1	over	ND	ND	ND	ND	0,00	2,63	1,16	0,648
S13	4575	F3	over	0,00	3,42	1,38	0,668	0,00	2,83	1,18	0,671
S14	9180	F3	over	0,00	3,98	2,00	0,707	ND	ND	ND	ND
S15	6815	F4	under	3,34	-2,40	0,81	0,637	ND	ND	ND	ND

These peaks were selected according to their False Discovery Rate (FDR), their Fold Change (FC), their AUC ROC and the SAM test. The peak S1 and S13 were significant both in EP and LP; S2, S3, S4, S14 and S15 were significant in EP; S5, S6, S7, S8, S9, S10, S11 and S12 were significant in LP. ND: Non determined, only for non significant values.

**Figure 3:**
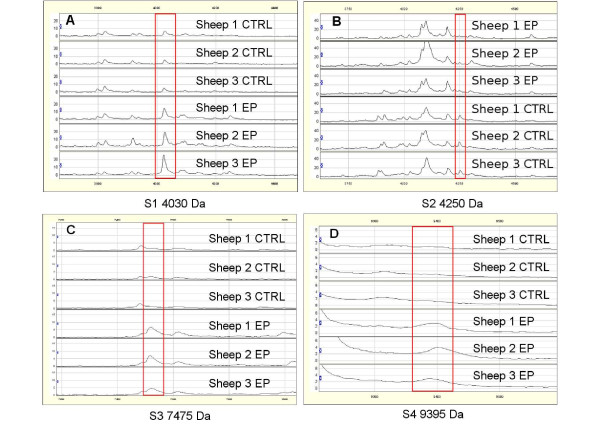
**Representative SELDI-TOF MS protein profiles of serum samples from three healthy animals and three scrapie sheep**. (A) Representation of the 4030 Da protein (S1) contained in F4. (B) Representation of the 4250 Da (S2) protein contained in F6. (C) Representation of the 7475 Da (S3) protein contained in F3. (D) Representation of the 9395 Da (S4) protein contained in F1.

**Figure 4:**
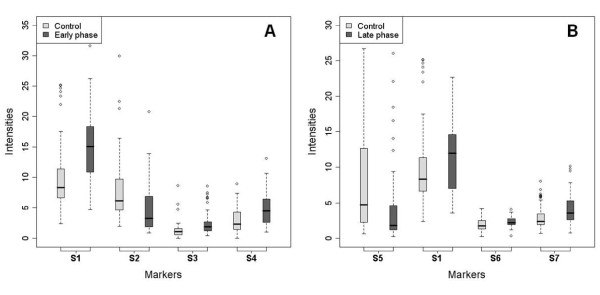
**Representative distribution of the intensities of selected biomarkers in the sheep populations**. (A) Distribution of biomarkers S1 to S4 in control and EP sheep populations. (B) Distribution of biomarkers S1 and S5 to S7 in control and LP sheep populations. The thick line in the boxes indicates the median value of intensities for each population.

First, 55 serum samples from 7-9 month/old animals in the EP of scrapie, which had been classified as PrP^VRQ^/PrP^VRQ^, were compared to 65 serum samples from healthy PrP^ARR^/PrP^ARR ^sheep. The EP of scrapie infection is very important for diagnostic purposes as the clinical symptoms are not apparent yet and the pathological agent PrP^Sc ^is moving from lymphoid organs to the brain. Seven peaks were found to be differentially expressed in EP animals in comparison to controls and four of them (i.e., S1 4030 Da, S2 4250 Da, S3 7475 Da and S4 9395 Da) were considered to be the best candidates as biomarkers of EP scrapie (see SELDI profiles in Figure 3, and Table 2). Specifically, three of these peaks (S1, S3 and S4) were over-expressed and one (S2) was under-expressed in EP sheep compared to controls. Their significant SAM values were ≥ 3.40 and the fold change was ≥ 1.75 or 0.5 with a null q-value. Moreover, the biomarker accuracy test (ROC) of the four peaks showed an AUC > 0.717 with the following individual values: 0.771 (S1), 0.735 (S2), 0.741 (S3) and 0.717 (S4). The distribution of the intensity values for the S1 to S4 biomarkers in control and EP sheep populations (Figure 4A) confirmed that S1, S3 and S4 were significantly over-expressed and S2 under-expressed in EP sheep in comparison to healthy controls.

Then, 43 serum samples from 13-19 month/old PrP^VRQ^/PrP^VRQ ^sheep with LP scrapie were compared to the 65 serum samples from healthy, PrP^ARR^/PrP^ARR ^animals. In LP scrapie, animals develop clinical symptoms (fear, nervousness, ataxia of the hind limbs, nibbling and licking) as the central nervous system is affected. Among the ten peaks differentially expressed in LP animals, four (i.e., S1 4030 Da, S5 3895 Da, S6 7690 Da and S7 9425 Da) were chosen as putative LP biomarkers (see Table 2 for statistical data). Three peaks (S1, S6 and S7) were over-expressed and one peak (S5) was under-expressed in LP sheep compared to controls. Their significant SAM values were ≥ 1.44 and the fold change was ≥ 1.26 or 0.38. Moreover, the biomarker accuracy test (ROC) of the four peaks showed an AUC > 0.617 with the following individual values: 0.617 (S1), 0.702 (S5), 0.659 (S6) and 0.693 (S7). The distribution of the intensity values of S1, S5, S6 and S7 (Figure 4B) confirmed the over-expression of markers S1, S6, S7 and the under-expression of marker S5 in LP sheep in comparison to healthy controls.

In conclusion, 7 peaks were found to significantly differentiate EP from control or LP animals and 10 peaks to significantly discriminate LP from control or EP animals. All together 15 different peaks were selected and 2 peaks (S1 and S13) were found to be significant in both phases of the disease.

### EP and LP biomarkers panel best combination

**Figure 5:**
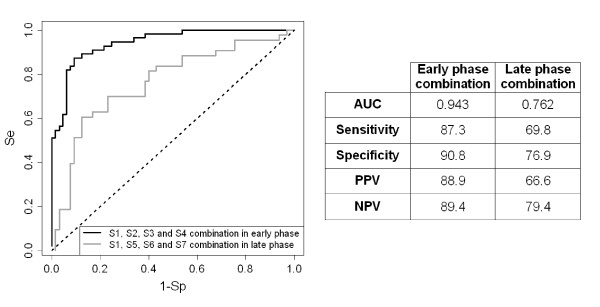
**mROC graph representations of sensitivities and specificities of the combination of EP (black curve) or LP (grey curve) biomarkers**. The mROC linear equations for decision rule are *Z*_EP _= -1.547 × [S2*] +0.466 × [S3*] + 0.661 × [S4*] + 1.238 × [S1*]; Z_LP _= 0.295 × [M1*] + 0.397 × [M5*] + 0.176 × [M6] + 0.399 × [M7*] * with λ_S1_= 0.05, λ_S2 _= -0.17, λ_S3 _= 0.32, λ_S4 _= 0.44, λ_S5 _= -0.18, λ_S7 _= 0.01 where λ is the coefficient of Box-Cox transformation: (S* = (S^λ^-*1*)/λ)

The previous data indicate that none of the selected biomarkers is sensitive and specific enough to be used as a single signature biomarker of scrapie in EP or in LP. We thus used the mROC program to test whether the combination of the four best EP (S1-S2-S3-S4) or of the four best LP (S1-S5-S6-S7) biomarkers could improve their diagnostic accuracy (Figure 5). The analysis of the ROC curve for the EP combination gave a better AUC (0.943) compared with the AUCs of the individual biomarkers and this combination showed a sensitivity of 87.3% and a specificity of 90.8%. Conversely, the panel of LP biomarkers reached only a sensitivity of 69.8% and a specificity of 76.9%; however, its AUC of 0.762 was better than the one of the single biomarkers, confirming that the combined set of LP biomarkers was more reliable than the individual biomarkers.

AdaBoost and SVM were also used to evaluate the potential diagnostic characteristics of our set of biomarkers. The Se and Sp values obtained with AdaBoost and SVM were comparable to those obtained with mROC (Table 3). Specifically, when control sheep were compared to EP sheep all methods generated results closed to the mROC values with an average specificity and sensitivity of 88.1% and 87.3% respectively. When control sheep were compared to LP sheep, the obtained Se and Sp values were more variable with an average specificity of 77.2% and an average sensitivity of 69.6%. In this case, Adaboost (accuracy = 0.780) seemed to perform better than SVM (accuracy = 0.729). We investigated the possible correlation of S1 to S7 biomarkers with sex and the corresponding *p*-values were obtained according to one-way ANOVA with a no significant value of more than 0.05.

**Table 3:** Diagnostic performances of biomarkers combination using three different classifiers

Classifiers	Healthy (n = 65) Vs EP (n = 55)			Healthy (n = 65) Vs LP (n = 43)		
	Accuracy	Se	Sp	Accuracy	Se	Sp
mROC	0,891	87,3	90,8	0,741	69,8	76,9
AdaBoost	0,867	86,6	86,8	0,780	82,5	71,1
SVM	0,873	88,0	86,8	0,729	56,5	83,7

The different classifiers used were: SVM (Kernel = radial, gamma = 0.2); Adaboost (weak learner: Decision stump, iteration = 100, weight treshold_EP _= 50, weight treshold_LP _= 85) and mROC.

### Analysis of the appearance of serum biomarkers in VRQ/VRQ Cheviot TSE free sheep

**Figure 6:**
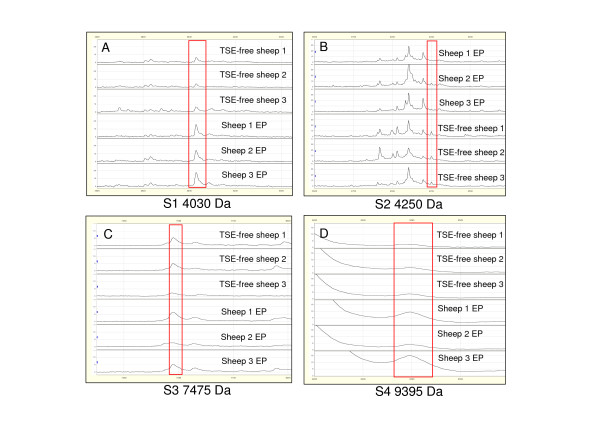
**Representative SELDI-TOF MS protein profiles of serum samples from three negative control TSE free sheep and three EP scrapie sheep**. (A). Representation of the 4030 Da protein (S1) contained in F4. (B) Representation of the 4250 Da (S2) protein contained in F6. (C) Representation of the 7475 Da (S3) protein contained in F3. (D) Representation of the 9395 Da (S4) protein contained in F3.

To test whether the 4 best combined EP biomarkers (S1 to S4) and the 4 best combined LP biomarkers (S1, S5, S6 and S7) presented in table 2 could be significant in another sheep, we analyzed serum samples from 3 VRQ/VRQ Cheviot TSE free sheep (negative controls). Sera were fractionated and analyzed in the 2-20 kDa range using CM10 Proteinchips^®^. The SELDI-TOF MS protein profiles of fractionated serum from negative controls (n = 3) and infected EP sheep (n = 3) were significantly different, focusing independently on each biomarker of interest (Figure 6). Minor or no differences were observed comparing the LP biomarkers on SELDI-TOF MS protein profiles of fractionated serum samples from negative control sheep (n = 3) with those from LP sheep (n = 3). These results show that the best combination of EP biomarkers could be significant comparing serum samples collected from animals from different laboratories. Furthermore, these results suggest that the EP signature pattern is correlated to the disease and not to the genotype. Still, an increased number of serum samples from TSE free sheep is necessary to further confirm theses observations.

### Analysis of the appearance of serum biomarkers in hamsters infected with the 263 K scrapie strain

**Figure 7:**
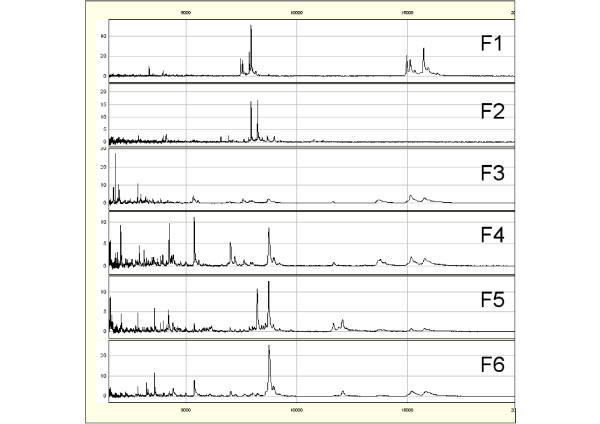
**Representative fractionated serum SELDI-TOF MS protein profiles of Syrian hamster infected with the 263 K scrapie strain (day 29 post-infection)**: F1 (pH9), F2 (pH7), F3 (pH5), F4 (pH4), F5 (pH3), F6 (organic wash).

**Figure 8:**
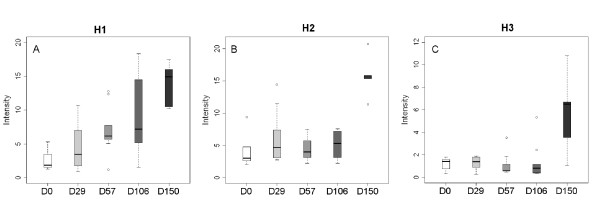
**Representative distribution of the intensities of selected biomarkers in Syrian hamsters infected with the 263 K scrapie strain**: (A) H1 (12 030 Da), (B) H2 (3895 Da) and (C) H3 (7555 Da) at day 0, day 29, day 57, day 106, day 150 post-infection.

To test whether the 15 biomarkers (S1 to S15) presented in table 2, including biomarkers specific for EP and LP phase of scrapie could be detected in another species using the same technology, we infected ten Syrian hamsters, in which the course of the disease is much more rapid than in sheep, with the 263 K scrapie strain and collected serum at different time points (day 0, 29, 57, 106 and 150 post-infection) during disease progression. As with the sheep samples, hamster sera (diseased and healthy controls) were fractionated and each anion-exchange fraction (pH9, pH7, pH5, pH4, pH3 and the organic wash) was analyzed in the 2-20 kDa range using CM10 Proteinchips^® ^under identical conditions and in the same day. The SELDI-TOF MS protein profiles of serum from Syrian hamsters were different from those of sheep (Figure 7 and compare with Figure 2). Nevertheless, the mass of three of the fifteen sheep candidate biomarkers could be detected in the hamster protein profile: S10 (named H1, found in F6 in the hamster model), S5 (named H2, found in F2 in the hamster model) and S12 (named H3, found F1 in the hamster model). Their intensity was followed throughout the course of the disease and two different behaviors could be observed. The intensity of peak H1 increased early after infection and continued to grow in a regular way (Figure 8A). Conversely, the intensity of peak H2 and H3 remained stable and dramatically increased only at the very end of the LP of disease (almost before the death of the animals) (Figures 8B and 8C). These results suggest that also in the hamster model of scrapie it is possible to distinguish between proteins which are differentially expressed in the EP of the disease (H1) in comparison to healthy controls and proteins which are characteristic of the LP of infection (H2 and H3).

### Identification and validation of S10 biomarker in sheep

**Figure 9:**
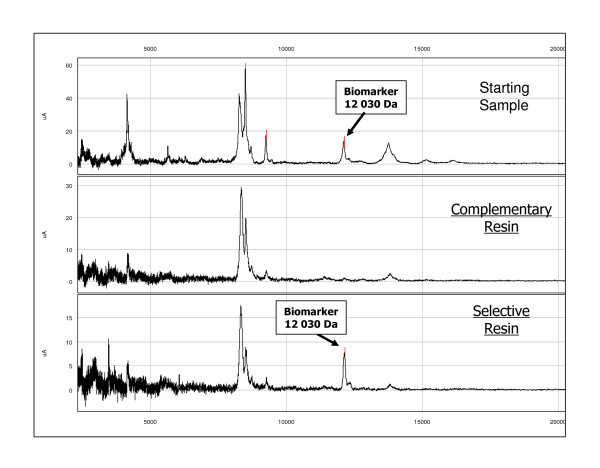
**Selection of complementary and selective resins for the 12 030 Da biomarker**.

**Figure 10:**
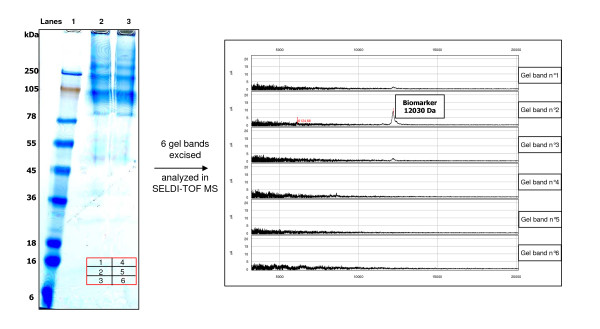
**SDS-Page separation and SELDI-TOF MS analysis of the purified 12 030 Da biomarker**: 0.5 ml of F6 fraction after overload on 10 μl selective resin was used.

**Figure 11:**
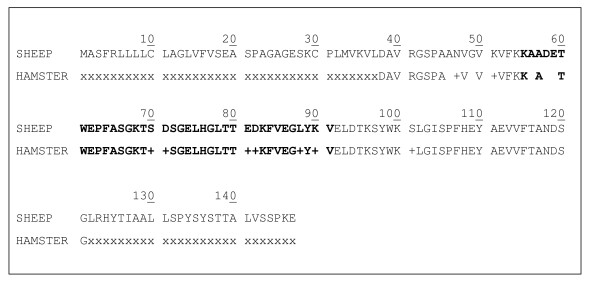
**Ovine and hamster transthyretin sequences**: peptides identified by LC-MS/MS are highlighted. (+) represent amino acids with similar physico-chemical properties.

We focused our interest on the H1 peak which could be considered as an early biomarker of disease in the hamster model. H1 had a mass of 12 030 Da which corresponded to that of peak S10 in sheep. However, while in the hamster model H1 was over-expressed, in sheep S10 was down-regulated and it was classified as a marker of LP scrapie (Table 2). With the aim of understanding the reason of this discrepancy, we decided to identify in sheep serum the protein composition of this peak. We first performed purification and enrichment (see Materials and Methods) of the 12 030 Da biomarker obtained from selected resins (Figure 9). The 12 030 Da biomarker was successfully enriched and amplified after scaling up by 100 folds the sample load on selective resins. Step gradient elution using optimal conditions also improved markers purity. The final step of the purification consisted in SDS-PAGE separation and SELDI-TOF MS analysis of the selected 12 030 Da band which was visible after colloidal blue staining thanks to the 100 fold scale-up amplification (Figure 10). Finally, using LC-MS/MS analysis, the S10 peak was identified as a major fragment (37 amino acids) of the transthyretin monomer (147 amino acids, P12303). The corresponding peptides are described in Figure 11 and the sequence coverage of the protein is 29.4%. The transthyretin protein in hamster (84 amino acids, B3VTM4) presents 94% of similarities with the sheep one and 91% considering the fragment of interest corresponding to S10 biomarker. Furthermore, we investigated the reproducibility of S10 under-expression and H1 over-expression by western blot analysis (see additional file 1: Transthyretin western blot analysis in serum from 3 LP sheep (VRQ/VRQ) and 3 healthy sheep (ARR/ARR); additional file 2: Transthyretin western blot analysis in serum (F6) from 2 Syrian hamsters at different kinetic points of the scrapie 263 K infection). The results showed an under-expression of the transthyretin monomer, dimer and tetramer (circulating forms of transthyretin) in serum from healthy sheep comparing to the same isoforms in serum from LP sheep. In hamsters infected by scrapie, we found the expression of the monomer isoform increasing with the time of infection. We also determined the transthyretin protein level in serum from healthy sheep (n = 20), EP sheep (n = 10) and LP sheep (n = 11) (see additional file 3: Quantitative analysis of serum levels of transthyretin). Total transthyretin protein concentration is significantly decreased in LP sheep (fold change median = 1.46 and *p*-value < 0.001) whereas it is not in EP sheep (fold change median = 1.11 and *p*-value > 0.1). All these observations correlate to the SELDI-TOF MS analysis. The curve representations of total ovine transthyretin concentrations fluctuation in healthy and pathological sheep indicate that transthyretin under-expression is not correlated with the age.

## Discussion

In this study, we report the first analysis of potential biomarkers in serum of sheep during the first 7 to 10 months of scrapie infection. Our results indicate that we have detected a biomarker profile which could be used to diagnose scrapie in sheep with no apparent symptoms during the incubation phase of the disease. This is important as access to serologic markers can avoid invasive acts as biopsy or lumbar punction; however, complementary research is needed to confirm the real relevance of these proteins as TSEs biomarkers. Particularly, age matching is not fully balanced since it was difficult to find a large cohort of young control sheep. A validation study with age-matched animals will permit to confirm the robustness of the results. The main bottle-neck of SELDI-TOF MS profiling technology is the purification and the identification of individual proteins, therefore we need to concentrate our resources on biomarkers that are most likely to be biologically significant, such as the 4-protein signature we detected in EP sera. Conversely, the specificity and sensitivity of the combination of LP markers is not sufficient enough to exploit them further.

The kinetic study of the proteomic content of sera from hamsters infected with the 263 K scrapie strain allowed the detection of three differentially expressed peaks (H1, H2, H3). H1 increased early and regularly during the course of the disease, whereas H2 and H3 increased suddenly at very end of the infection process. None of these biomarkers was found to increase in control, not infected hamsters. Interestingly, S10 and H1 have the same molecular mass, but their intensities vary differently in sheep and in hamsters along time. S10 was then identified as a fragment of the transthyretin monomer. Transthyretin or pre-albumin is a homo-tetramer glycoprotein synthesized by liver and present in plasma, serum and cerebrospinal fluid. It has a molecular mass of 55 kDa and is the main thyroxin and vitamin A transporter. Transthyretin is an early marker of under-nutrition; its plasma level is decreased in case of liver failure, inflammatory syndrome and increased in case of chronic renal insufficiency. Transthyretin was already quoted in literature as a molecule associated with neurological disorders like multiple sclerosis [57], amyloid polyneuropathy [58-60] and TSEs [61]. It has been previously described as a Creutzfeldt-Jakob disease biomarker detectable in the cerebrospinal fluid [62]. Furthermore, several proteins linked to neurodegenerative diseases, such as amyloid beta, tau, prions and transthyretin, were found to be glycated in patients, and this is thought to be associated with increased protein stability through the formation of crosslinks that stabilize protein aggregates [63]. A proteic characterization of the transthyretin fragment found discriminant in our study can provide useful informations for TSEs diagnostic. The molecular mass of the transthyretin monomer is 15.7 kDa; therefore by SELDI-TOF MS we detected a major fragment of the monomer. In scrapie sheep, its intensity decreased with the disease, whereas it increased in infected hamsters. Furthermore, sample handling can play a role in proteomics variations [64]. Finally, since in hamsters scrapie is not a naturally occurring disease, different pathological mechanisms, and hence different protein signatures, could play a role in the development of the disease. Unfortunately, due to volume limitations, we could not identify the 12 030 Da SELDI peak in hamster serum for cross-validation. The transthyretin analysis done by western blot and serum level quantification confirmed the SELDI-TOF MS results. In a recent study, a training set of biomarkers has been established in brain homogenate samples from a murine model infected by the ME-7 scrapie strain [65]. Two of the biomarkers found discriminant in the infected animals compared to controls may correspond in m/z to the biomarkers S12, H3 (7555 Da) and S14 (9180 Da). Their fluctuation is inversely correlated with S12 and S14, confirming that depending on the model and the time course study, biomarkers can be significantly down or up regulated and that protein expression in TSE disease exist across different species. The biomarkers we identified can be used as a target of development of future immunobased assay more suitable for veterinary or clinical analysis and compatible with blood screening.

## Conclusion

Currently, the quest of non invasive TSEs biomarkers remains important and the development of a rapid, sensitive, specific, *ante-mortem *test is still needed. Indeed, a recent report detailed that prions adhere to soil minerals and remain infectious [66] such that unidentified environmental reservoirs of infectivity contribute to the natural transmission of prion diseases in sheep, deer and elk. Although the pandemic infection is now minimized, we need to remain vigilant to prevent a new crisis. The development of non-prion protein biomarkers for TSEs has been reported recently due to the advances in post-genomic technologies. However, more work needs to be done to confirm the specificity and sensitivity of bioassays combining the identified biomarkers. The remaining steps will be to design specific probes against biomarkers and optimize bioassay condition. Then, large scale validation would be affordable. These steps are mandatory to envision a future peripheric TSE bioassay.

## List of abbreviations

(**PrP^Sc^)**: Pathological Prion Protein; (**PrP^c^**): Normal Prion Protein; (**TSEs**): Transmissible Spongiform Encephalopathies; (**2-DE**): Bi-dimensionnal electrophoresis; (**SELDI-TOF MS**): Surface Enhanced Laser Desorption/Ionization-time of flight-mass spectrometry; (**EP**): Early Phase; (**LP**): Late Phase; **(μL) **: Microliters; (**mL**): Milliliter; (**Se**): Sensitivity; (**Sp**): Specificity; (**SAM**): Significance Analysis of Microarray; (**SVM**): Support Vector Machine; (**FT**): Flow-Through fraction; (**AUC**): Area Under the Curve; (**ROC**): Receiving Operating Characteristics; (**Da**): Dalton; (**LC-MS/MS**)Liquid Chromatography-mass spectrometry; (**MALDI-TOF MS**): Matrix Assisted Laser Desorption Ionization-time of flight-mass spectrometry; (**F**): Fraction

## Competing interests

The authors declare that they have no competing interests.

## Authors' contributions

All authors read and approved the final manuscript. IBM and CMG conceived and designed the study. OA has provided all sheep serum samples with secure diagnostic and advised IBM on intellectual content. IBM, CMG and GV performed the experimentations relatives to the hamster's infection and the corresponding kinetic. IBM analyzed samples and collected the results. IBM and LG performed the experimental steps to identify the S10 biomarker. NS and FM carried out all statistical analysis involved in the study. IBM wrote the manuscript and NS, FM, LG and CMG contributed to drafting the manuscript.

## Supplementary Material

Additional file 1 **Transthyretin western blot analysis in serum from 3 LP sheep (VRQ/VRQ) and 3 healthy sheep (ARR/ARR)**. Fifty μg of proteins from serum samples have migrated on a SDS-Page acrylamide 12% electrophoretic gel (lines 1 to 3: pathological VRQ/VRQ sheep; lines 4 to 6: healthy ARR/ARR sheep; border line control "Ctl": 100 ng of Recombinant full length Human Prealbumin, amino acids 21-147, 13,8 kDa abcam n°92931). The transthyretin signal is revealed by a primary polyclonal antibody from rabbit (abcam n°16006; immunogen = prealbumin isolated from human plasma; reacts with human, sheep) used at 1 μg/mL in 0.1% PBS-Tween/2% milk and a secondary antibody coupled with HRP diluted 1/80 000 in 0.1% PBS-Tween/2% milk. The molecular weight standards are mentioned in kilo Dalton (Bio-Rad). Click here for file

Additional file 2 **Transthyretin western blot analysis in serum (F6) from 2 Syrian hamsters at different kinetic points of the scrapie 263 K infection**. Ten μg of proteins from serum samples (F6) from infected Syrian hamsters have migrated on a SDS-Page acrylamide 12% electrophoretic gel (lines 2 to 5: kinetic points J0, J29, J57, J106 of hamster n°1; l ines 6 to 9: kinetic points J29, J57, J106, J150 of hamster n°2; border line control "Ctl": 100 ng of recombinant transthyretin protein). The transthyretin signal is revealed by a primary polyclonal antibody from rabbit used at 1 μg/mL in 0.1% PBS-Tween/2% milk and a secondary antibody coupled with HRP diluted 1/80 000 in 0.1% PBS-Tween/2% milk. The signal corresponding to the transthyretin fragment is localized on the gel between the molecular weigth proteins 10 and 25 kDa. Click here for file

Additional file 3 **Quantitative analysis of serum level of transthyretin**. Box plot representation of total ovine transthyretin concentrations in serum from healthy sheep (ARR/ARR), EP sheep (VRQ/VRQ) and LP sheep (VRQ/VRQ) populations. The median fold change is 1.11 comparing the median concentration values of serum from healthy sheep *versus *EP sheep and 1.46 comparing the median concentrations values of serum from healthy sheep *versus *LP sheep. The median concentration value of transthyretin in serum from healthy sheep is 252.80 μg/mL, in EP sheep 227.60 μg/mL and in LP sheep 172.80 μg/mL. Kaleidagraph 4.0 software was used to calculate the *p *value (Wilcoxon test) and to present the boxplot graphs. Curve representation of total ovine transthyretin concentration fluctuation in serum from healthy sheep (ARR/ARR). Curve representation of total ovine transthyretin concentration fluctuation in serum from pathological sheep (VRQ/VRQ). Click here for file
