# Supplementary material for: Ovine serum biomarkers of early and late phase scrapie
Source: BMC Vet Res. 2010 Nov 2;6:49. doi: 10.1186/1746-6148-6-49 (PMC2988006; doi:10.1186/1746-6148-6-49)
Supplement: Additional file 1 — Transthyretin western blot analysis in serum from 3 LP sheep (VRQ/VRQ) and 3 healthy sheep (ARR/ARR). Fifty μg of proteins from serum samples have migrated on a SDS-Page acrylamide 12% electrophoretic gel (lines 1 to 3: pathological VRQ/VRQ sheep; lines 4 to 6: healthy ARR/ARR sheep; border line control "Ctl": 100 ng of Recombinant full length Human Prealbumin, amino acids 21-147, 13,8 kDa abcam n°92931). The transthyretin signal is revealed by a primary polyclonal antibody from rabbit (abcam n°16006; immunogen = prealbumin isolated from human plasma; reacts with human, sheep) used at 1 μg/mL in 0.1% PBS-Tween/2% milk and a secondary antibody coupled with HRP diluted 1/80 000 in 0.1% PBS-Tween/2% milk. The molecular weight standards are mentioned in kilo Dalton (Bio-Rad). [file 1746-6148-6-49-S1.PDF]

<u>Additional file 1</u>: Transthyretin western blot analysis in serum from 3 LP sheep (VRQ/VRQ) and 3 healthy sheep (ARR/ARR).

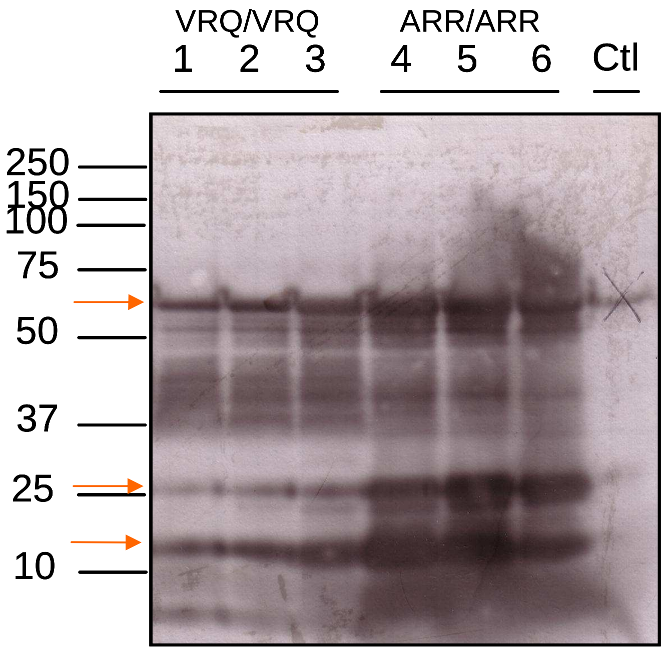

Fifty µg of proteins from serum samples have migrated on a SDS-Page acrylamide 12% electrophoretic gel (lines 1 to 3: pathological LP VRQ/VRQ sheep; lines 4 to 6: healthy ARR/ARR sheep; border line control "Ctl": 100 ng of Recombinant full length Human Prealbumin, amino acids 21-147, 13,8 kDa abcam n°92 931). The transthyretin signal is revealed by a primary polyclonal antibody from rabbit (abcam n°16006; immunogen = prealbumin isolated from human plasma; reacts with human, sheep) used at 1 µg/mL in 0.1% PBS-Tween / 2% milk and a secondary antibody coupled with HRP diluted 1/80 000 in 0.1% PBS-Tween / 2% milk. The molecular weight standards are mentioned in kiloDalton (Bio-Rad).
