# Supplementary material for: Ovine serum biomarkers of early and late phase scrapie
Source: BMC Vet Res. 2010 Nov 2;6:49. doi: 10.1186/1746-6148-6-49 (PMC2988006; doi:10.1186/1746-6148-6-49)
Supplement: Additional file 2 — Transthyretin western blot analysis in serum (F6) from 2 Syrian hamsters at different kinetic points of the scrapie 263 K infection. Ten μg of proteins from serum samples (F6) from infected Syrian hamsters have migrated on a SDS-Page acrylamide 12% electrophoretic gel (lines 2 to 5: kinetic points J0, J29, J57, J106 of hamster n°1; l ines 6 to 9: kinetic points J29, J57, J106, J150 of hamster n°2; border line control "Ctl": 100 ng of recombinant transthyretin protein). The transthyretin signal is revealed by a primary polyclonal antibody from rabbit used at 1 μg/mL in 0.1% PBS-Tween/2% milk and a secondary antibody coupled with HRP diluted 1/80 000 in 0.1% PBS-Tween/2% milk. The signal corresponding to the transthyretin fragment is localized on the gel between the molecular weigth proteins 10 and 25 kDa. [file 1746-6148-6-49-S2.PDF]

Additional file 2: **Transthyretin western blot analysis in serum (F6) from 2 Syrian hamsters at different kinetic points of the scrapie 263K infection.**

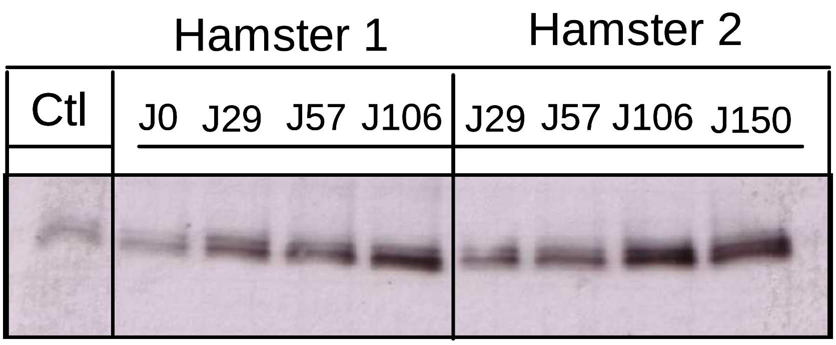

Ten µg of seric proteins (F6) from infected Syrian hamsters have migrated on a SDS-Page acrylamide 12% electrophoretic gel (lines 2 to 5: kinetic points J0, J29, J57, J106 of hamster n°1; lines 6 to 9: kinetic points J29, J57, J106, J 150 of hamster n°2; border line control “Ctl”: 100 ng of Recombinant full length Human Prealbumin, amino acids 21-147, 13,8kDa abcam n°92931). The transthyretin signal is revealed by a primary polyclonal antibody from rabbit (abcam n°16006; immunogen = prealbumin isolated fro m human plasma; reacts with human, sheep) used at 1 µg/mL in 0.1% PBS-Tween / 2% milk and a secondary antibody coupled with HRP diluted 1/80 000 in 0.1% PBS-Tween / 2% milk. The signal corresponding to the transthyretin fragment is localized on the gel between the molecular weigth proteins 10 and 25 kDa.
