# Supplementary material for: Ovine serum biomarkers of early and late phase scrapie
Source: BMC Vet Res. 2010 Nov 2;6:49. doi: 10.1186/1746-6148-6-49 (PMC2988006; doi:10.1186/1746-6148-6-49)
Supplement: Additional file 3 — Quantitative analysis of serum level of transthyretin. Box plot representation of total ovine transthyretin concentrations in serum from healthy sheep (ARR/ARR), EP sheep (VRQ/VRQ) and LP sheep (VRQ/VRQ) populations. The median fold change is 1.11 comparing the median concentration values of serum from healthy sheep versus EP sheep and 1.46 comparing the median concentrations values of serum from healthy sheep versus LP sheep. The median concentration value of transthyretin in serum from healthy sheep is 252.80 μg/mL, in EP sheep 227.60 μg/mL and in LP sheep 172.80 μg/mL. Kaleidagraph 4.0 software was used to calculate the p value (Wilcoxon test) and to present the boxplot graphs. Curve representation of total ovine transthyretin concentration fluctuation in serum from healthy sheep (ARR/ARR). Curve representation of total ovine transthyretin concentration fluctuation in serum from pathological sheep (VRQ/VRQ). [file 1746-6148-6-49-S3.PDF]

<u>Additional file 3</u>: Quantitative analysis of serum levels of ovine transthyretin.

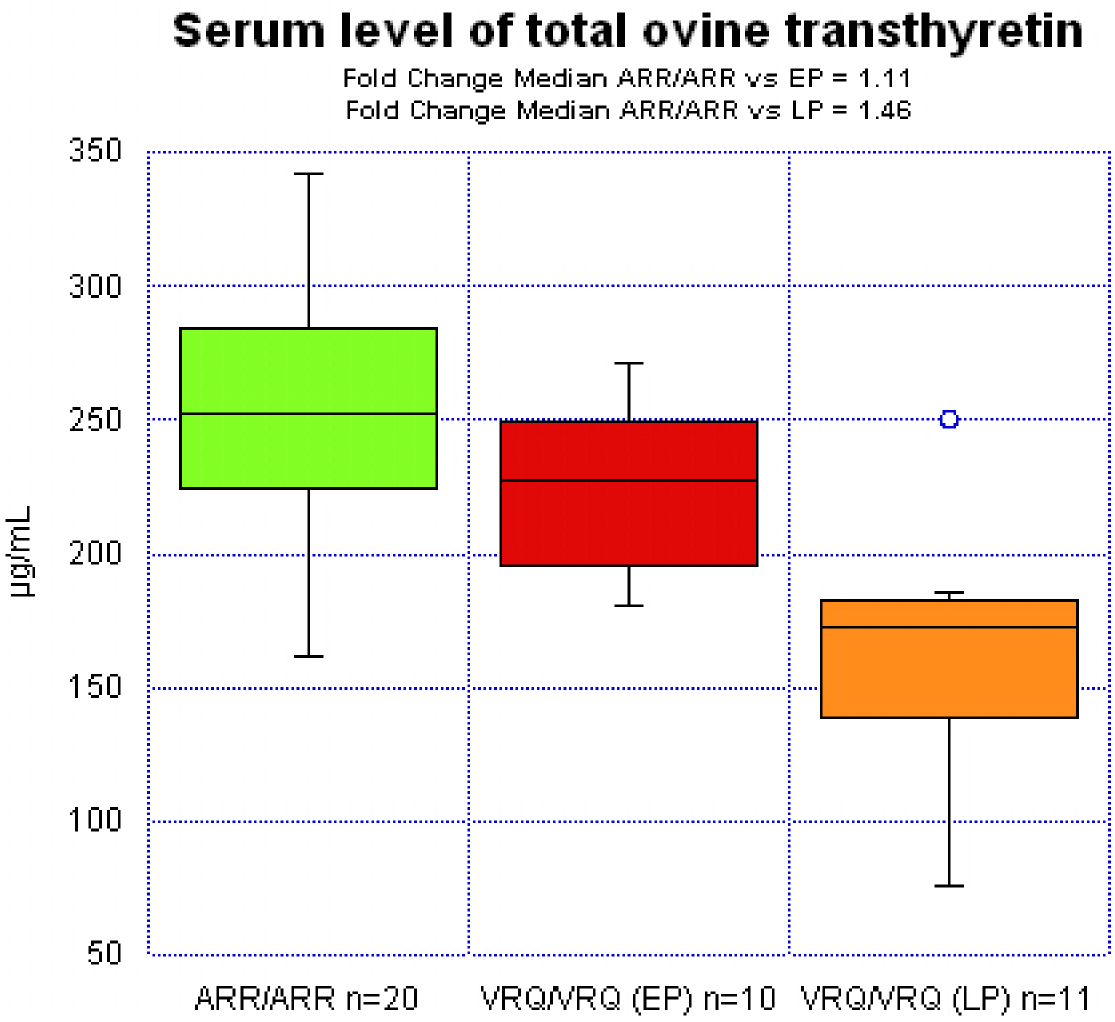

Box plot representation of total ovine transthyretin concentration in serum from healthy sheep (ARR/ARR), EP sheep (VRQ/VRQ) and LP sheep (VRQ/VRQ) populations. The median fold change is 1.11 comparing the median concentration values of serum from healthy sheep *versus* EP sheep and 1.46 comparing the median concentration values of healthy sheep *versus* LP sheep. The median concentration value of transthyretin in serum from healthy sheep is 252.80 µg/mL; in EP sheep 227.60 µg/mL and in LP sheep 172.80 µg/mL.

TTR ELISA kit (Cat # KA0495 V.02), Abnova, was used to quantify transthyretin in ovine serum. Kaleidagraph 4.0 software was used to calculate the *p* value (Wilcoxon test) and to present the boxplot graphs.

<u>Additional file 3</u>: Quantitative analysis of serum levels of ovine transthyretin.

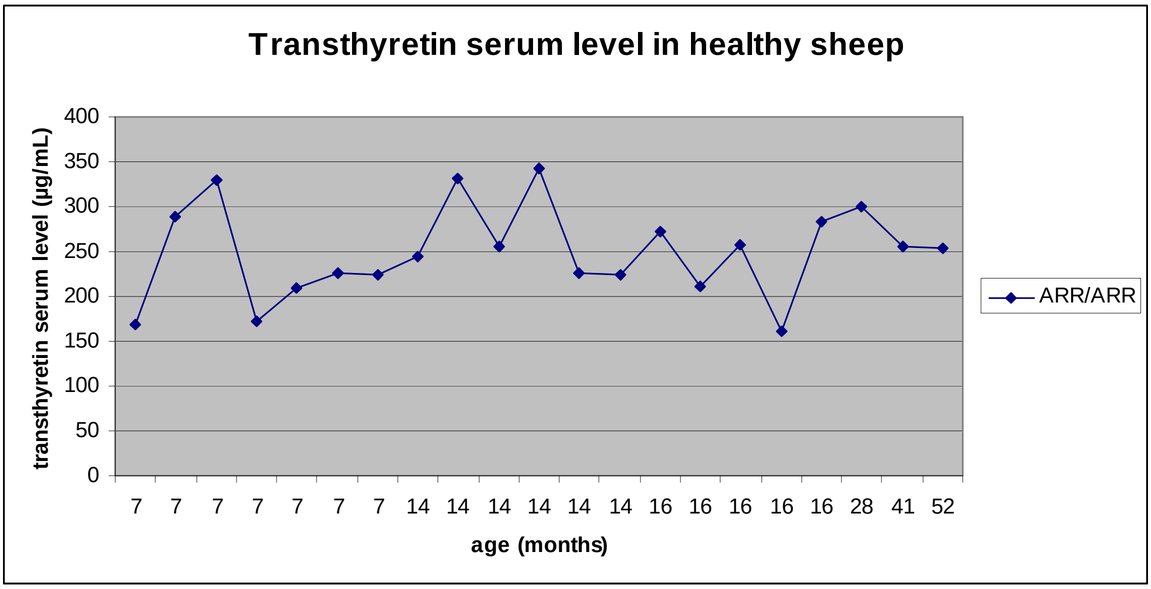

Curve representation of total ovine transthyretin concentration fluctuation in serum from healthy sheep (ARR/ARR).

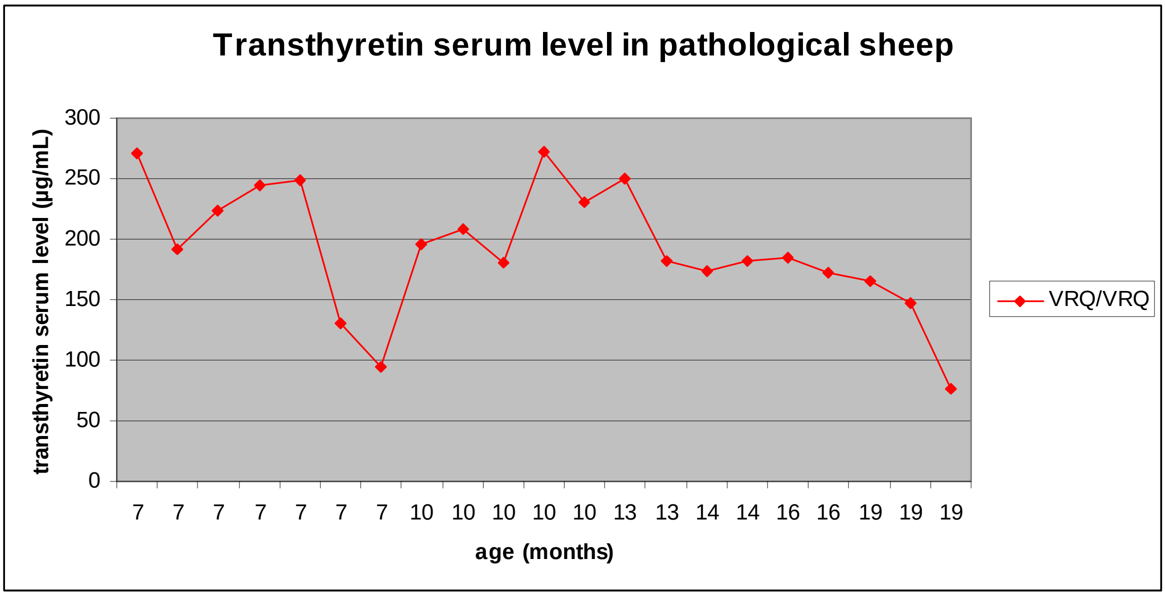

Curve representation of total ovine transthyretin concentration fluctuation in serum from pathological sheep (VRQ/VRQ).
